# Supporting novice designers design of digital touch

**DOI:** 10.1007/s10798-021-09722-1

**Published:** 2022-04-19

**Authors:** Carey Jewitt, Val Mitchell, Garrath Wilson, Lili Golmohammadi, Douglas Atkinson, Kerstin Leder Mackley, Sara Price

**Affiliations:** 1 UCL Knowledge Lab, Dept Culture, Communication & Media, IOE UCL’s Faculty of Education and Society, University College London, London, United Kingdom; 2 School of Design & Creative Arts, Loughborough University, Loughborough, United Kingdom

**Keywords:** Card-based resource, Technology, Touch, Digitally mediate touch, Design education, Design-based research

## Abstract

Digitally mediated touch is an emerging and significant area for technology and therefore for design and design education. However, the design of digital touch is a challenge, especially for novice designers, compounded by low awareness and understanding of the sociality of touch and the complexity of communicating felt sensations. This paper presents a qualitative study of a two-part educational intervention on the design of digital touch using a design-based research methodology. Findings are presented and discussed on the design challenges faced by novice designers in relation to touch and digital touch (the focus of part one of the intervention) and the development and piloting of the Designing Digital Touch (DDT) toolkit (the outcome of part two). The paper discusses how the toolkit can be used to foster and support novice designers to respond to the future facing complexity of digital touch design.

## Supporting novice designers design of digital touch

Touch is at the centre of a contemporary re-imagining of digital sensory communication (Oviatt et al., 2019) and an emergent area with significant design opportunities. Designing digital touch experiences can, however, be a challenge for designers, particularly novices. A challenge compounded by low awareness or understanding of the sociality of touch, the technical limitations of digitally mediated touch, and the complexity of communicating felt sensations. Digital touch refers to touch sensations delivered through computational technologies including co-located or remote touch communication, and might involve human-to-human, human-to-machine or machine-to-human touch. The term ‘novice designer’ refers to students, apprentice designers, as well as designers new to digital touch.

Design resources (e.g., card-based toolkits) can aid the education and design process of novice designers, providing information and inspiration to generate innovative concepts, and methods to support design (Roy & Warren, 2019) and are used in a wide range of design and design education settings. While some existing resources are broadly relevant to digital touch (e.g., embodiment, and the sensory), touch has received little focused attention within design.

This two-year study on the design of digital touch is motivated by the gap between the future-facing significance of this design space and the design resources available. It is an interdisciplinary collaboration between the InTouch team (academics in social science, human computer interaction, and design), and a leading UK design school. It explores how novice designers (UX design students) design of digitally mediated touch might be fostered and supported. Using a design-based research (DBR) approach (Anderson & Shattuck, 2012), a pragmatic, collaborative, flexible methodology, the study centres on a two-part design education intervention that aims to refine and improve practice (Wang and Hannafin, 2005: 6). Part one centred on the development of a UX module on designing digital touch. Part two involved the development of a card-based resource—the Designing Digital Touch (DDT) toolkit to support novice designers to integrate touch into their design process. The toolkit consists of a boxed-set of 190 cards (109 mm x 78 mm) mapped to six design stages; cards are colour coded to indicate a design stage, printed with a provocation, question or activity, and a reference code. They are designed to generate ideas, probe memories and provoke imaginations relevant to touch and the design process. The intervention was documented using design-based (e.g., prototyping, scenarios) and social science methods (e.g., field-notes, video observation). The paper presents and discusses the study findings and concludes by discussing the contribution and limitations of the study and intervention.

## Background

Two decades ago, touch technology was the stuff of science fiction (Shedroff & Noessel, 2012). Amidst a broader re-evaluation of the senses there is growing interest in digital touch within the social sciences (Jewitt et al., 2020), HCI (Hoggan, 2013), and design (Oviatt et al., 2019). This ‘turn to touch’ has the potential to re-shape the ‘what, who, how and when’ of touch experiences. While engaging with alternative futures is at the heart of technology education design (Gradwell, 1999), the future of digital touch raises complex questions and challenges for designers, especially novices.

There are significant differences between novice and experienced designers in how they represent and frame design problems (Björklund, 2013), the level of information that they seek, design strategies, and the use and understanding of prototypes (Deininger et al., 2017). This study is situated in studio practices (e.g. design-briefs, prototyping) which provides a bridge between academic and professional communities and contexts (Brandt et al., 2013). Touch and design are, however, embedded within tacit (implicit) knowledge and practices which can restrict access to new knowledge, ideas or approaches (Schindler, J., 2015), and require novices to engage with ideation beyond established practices of sketching or low-fidelity prototyping to articulate felt sensations. In addition, on their journey from novice to design practitioner students encounter many troublesome ‘threshold concepts’ which once grasped, can be transformative and are often integrative in creating new understandings and ways of thinking (Meyer & Land, 2006). For example, this may involve becoming tolerant of uncertainty and challenging the ideas of others (Osmond, Bull & Tovey, 2009), foregrounding design of experiences not usability, and ‘methodological fluency’ (e.g., recognising how to select an appropriate tool for a task) (Kharrufa & Gray, 2020).

In such complex design spaces, while design resources cannot fully compensate for the limited experience of novice designers (Bornoe, Bruun, & Stage, 2016), they have been shown to be a useful, convenient way to aid their design process. Design resources can help novices to generate innovative concepts, provide information, inspiration, and offer methods to support design (Roy & Warren, 2019), foster design dialogues, collaboration and argumentation (Brandt & Messerter, 2004), and facilitate the creation and reflective evaluation of design experiences (Fedosov et al. 2019).

Card-based design tools are sets of cards, similar to playing cards, with rules for their usage (e.g., card selection or arrangement). They usually consist of sub-sets of card categories that are shaped to address a specific aspect of a phenomena. Cards are used in different ways, for example, collecting and representing sources of inspiration, to gain an overview of concepts, a means of communication between designers and stakeholders/experts (Sanders, 2005) or to make a phenomena tangible (Brandt and Messerter,^2004^). A review of 155 card-based design tools (Roy & Warren, 2019) notes their ability to facilitate, externalize and communicate in the co-design process; support inspiration; add value to creative thinking; and provide, bridge and summarise information and methods. However, they can be complex, information heavy, and over-simplifying, and while a strength of card-based tools is that their physicality enables them to be handled, sorted and combined in creative ways, a weakness is they can be too ‘static’ (Roy & Warren, 2019).

Card-based design resources have been developed for a wide range of design domains (Roy & Warren, 2019), including embodiment (e.g., Somatic Tool), the digital (e.g., Inspiration Cards, Halskov & Dalsgård, 2006), and the sensory (e.g., Pink et al., 2020; Petreca et al., 2019). To date, however, touch and tactility have not been fully or explicitly brought into focus. The intervention reported in this paper contributes to this gap with the development of a design-resource to support digital touch design. It uses a design-based research (DBR) approach, which seeks to directly impact practice and advance understanding, theory or tools in ways that are meaningful for the study subjects—novice designers (Barab and Squire, 2004). DBR seeks to simultaneously advancing design, research and practice (The Design based Research Collective, 2003); researchers undertake design and research functions drawing on methods from both areas (here social science and design) in the form of ‘a hybrid methodology’ (Wang and Hannafin, 2005). That is they manage research processes, design and implement interventions to refine and improve practice. This approach is defined by a collaborative partnership between researchers and practitioners, a pragmatic focus on the design and testing of an intervention to improve practice, being situated in a real-world setting, a flexible mixed methodology and an integrative process involving iterative cycles of implementation, analysis and exploration, design, evaluation and reflection (Anderson & Shattuck, 2012; Wang and Hannafin, 2005).

## Methods

### Overview of approach and the intervention

This study uses a design-based research (DBR) approach (see Sect. 1) as it aligns well with the uncertain, fluid, future-facing design space of digital touch. The study is a collaboration between social science researchers and design education practitioners, in the form of a two-part intervention situated in a university design-education setting, which seeks to foster and support novice designers’ engagement with digital touch. As is typical of a DBR approach (Cobb et al., 2003), the findings and outcomes of part one of the intervention provided the focus of inquiry, design and development for part two. The study uses mixed methods in the form of iterative cycles of implementation, analysis and exploration, design, evaluation and reflection (Mckenney and Reeves, 2012).

Part one investigated how novice-designers conceptualise and work with digitally mediated touch. It took place over a nine-month period and implemented a digital touch focus to the BA UX design module using a student design-project-brief (see 3.1), introductory lecture, and two low-fidelity experience prototyping workshops. The participants design activity and development of digital touch design concepts was observed and analysed. These findings provided the analytical grounding (of the workshop interactions, prototypes and design concepts) and motivation for Part two of the intervention.

Part two of the intervention focused on the development and design of a card-based design resource—the Designing Digital Touch (DDT) toolkit. It was developed through iterative cycles of analysis and exploration, design, evaluation and reflection over 12 months, involving the social science researchers, design lecturers, and, in the piloting phase, UX design students. The initial design of the toolkit took place over a series of four one day team workshops involving the analysis of participants’ design concepts and prototyping, re-enactments and detailed walk-throughs, role play including use of the ‘stop-the-action’ technique (Price, Matthews and Wrigley, 2018), scenario building, brainstorming, and card proto-typing (see Sect. 4.1 and 4.2). This cyclical process centred on critical reflection and evaluation drawing on the teams’ contextual experiences of teaching design and researching touch.

The piloting and evaluation of the toolkit took place over two workshops (5–6). Participants used the toolkit to explore touch in the context of their individual major design projects rather than using the design brief—this reflected the structure of the MAUX (see Sect. 4.3).

Document sharing and toolkit prototyping activity took place between and after the above workshops.

### Research setting and collaborators

**Fig. 1:**
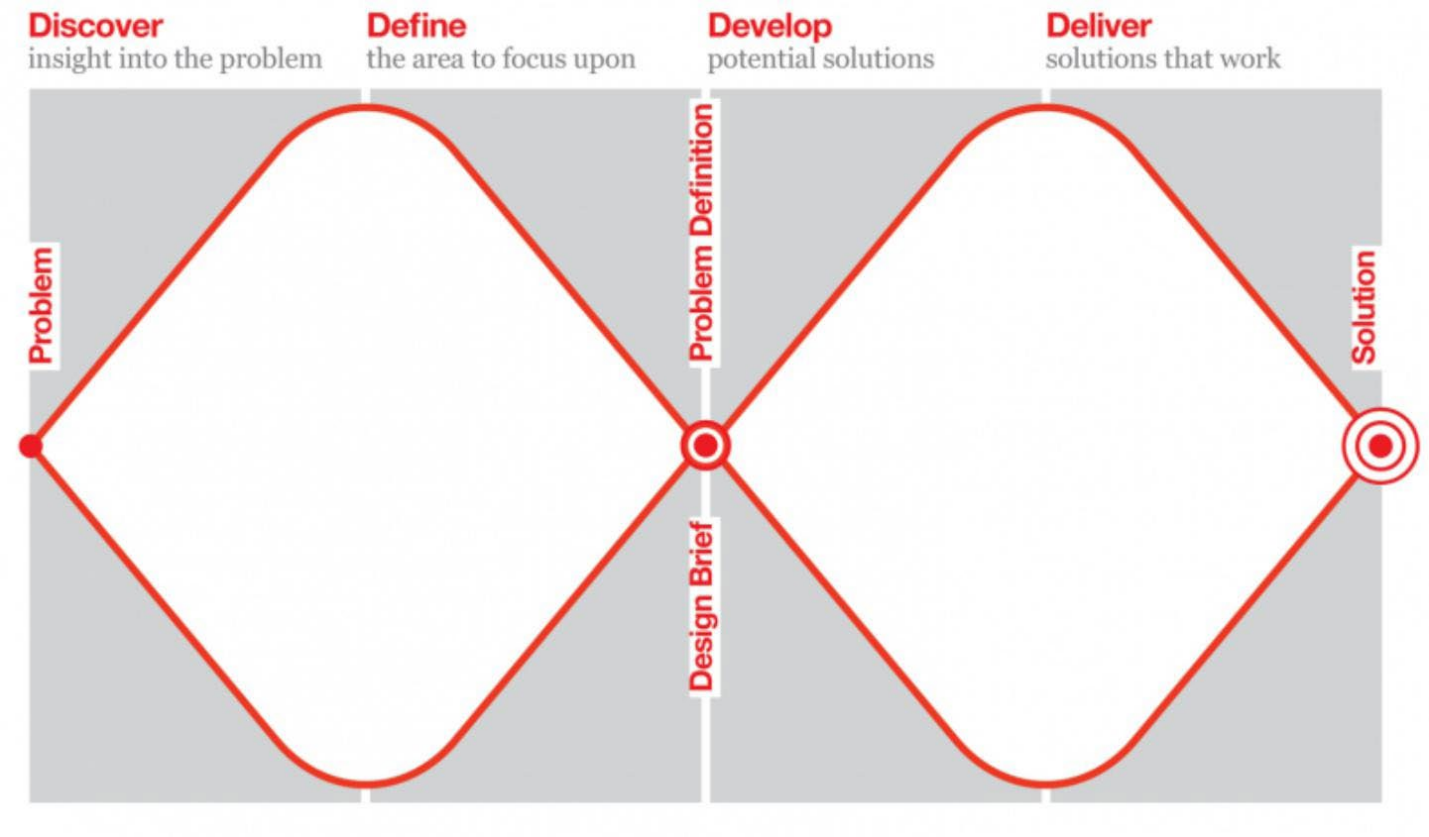
The Design Council ‘Double Diamond’ model (Design Council, 2005)

This study is a collaboration between the InTouchteam (with backgrounds in Sociology, HCI, and Design) and two Design lecturers at the School of Design and Creative Arts, <University name> a leading UK Design school. The research setting is a campus university, with a mix of home and international students, and a culture of applied and studio-based design pedagogy with expertise in human centred design, co-design, sustainable design, design futures, and user experience design. While the challenges of digital touch are across design areas, the study is situated within User-Experience design (UXD): being at the intersection of industrial design and digital design (Hassenzahl, 2013) UXD provides a good base for the exploration of digital touch. A primary model used in UXD is the UK Design Council ‘*Double Diamond*’ model (Design Council, 2005) which describes four key design stages common to product and service-centred innovation (see Fig. 1). This model was used within an iterative human centred design process which enabled it to be flexed to address touch, and the social, sensory, and speculative character of digital touch design futures.

The researchers’ relationships to the site differed: the InTouchteam were outsiders to the site and UX design, while the two design lecturers are insider-researchers. These differences were reflexively acknowledged and provided differing perspectives on the research and intervention development. At times the distinctions blurred between researcher, designer, participant-informant; this aligns with a DBR approach which positions researchers as necessary agents of change (Barab & Squire, 2004), and contributed to a shared understanding of the research and design context (Cobb et al., 2003).

### Participant recruitment and selection

Two cohorts of design students participated in the intervention. Recruitment was via a face-to-face introduction to the project and an information sheet. Participation was self-selecting and voluntary, it was made explicit that non-participation would not affect course experience or grading, and participants could withdraw from the study at any stage (participants could also participate in the workshop and not be a part of the research). Participants were offered different options regarding data collection and usage (e.g., consent to video record, and use for research only). All students chose to participate in the study.

A total of 85 participants were recruited—64 full-time undergraduate students (18 female, 46 male) in Cohort 1; and 21 full-time MA students (14 female, 7 male) in Cohort 2. Both cohorts were culturally diverse with participants from the USA and Americas, Asia/East-Asia, and Europe. Cohort 1 were year-two students, on the User-Experience (UX) Design module of the BA Industrial Design and Technology (IDT). This year-group was selected to ensure a shared understanding and competency in UX design. While we do not seek to compare the two cohorts, they are comparable in terms of their design experiences and their outputs: both received training from the same lecturers in a human centred design process framed by the Double Diamond.

During the workshops the participants worked in 6–10 small groups. To ensure a mix of depth and breadth in the data, three groups were designated as ‘primary groups’ purposefully sampled on the basis of the design lecturers’ assessment of the participant and the extent of their project engagement with touch. Data collection focused on these primary groups and ‘checked-in on’ the six secondary groups.

### Data collection and materials

DBR is an integrative approach and a mix of methods were used to document the intervention workshops and toolkit development including video recording, observation, photography, interviews and team discussion, and post-workshop field-notes. Table 1 presents an overview of the intervention activities, timeline and data collected.

**Table 1:** Overview summary of series of Intervention activities

Activity	Activity Length	Focus	Participants	Data
Dev. Brief & Module	M 1–6	Module on digital touch	Design Lecturers (DL) InTouch (IT)	Documents Field-notes
Lecture	M 7	Attuning students to touch	DL IT	PPT Field-note
Prototyping Session 1	M 8 3.5 h	Insights on novice designers’ touch conceptualisation & design process	DL IT Students (cohort 1) SC1	Video (7 h) Photos Documents Prototypes Field-notes
Prototyping Session 2	M 9 3.5 h	Insights on novice designers’ touch conceptualisation & design process	DL IT SC1	Video (7 h) Photos Documents Prototypes Field-notes
Analysis M 8–12				
Workshop 1	M 13 5 h	Discover: insights into the problem space	DL IT	Photos Documents Field-note
Workshop 2	M 15 6 h	Define: the area to focus on shape of Toolkit	IT	Photos Documents Field-note
Workshop 3	M 16 6 h	Develop: potential solutions—Iterate Toolkit	DL IT	Photos Documents Field-note
Workshop 4	M 16 6 h	Deliver: solutions that work-Iterate Toolkit	IT	Photo Documents Field-note
Soft-Launch	M 17	Introduce Toolkit	DL IT Students (Cohort 2) SC2	Photo Field-note
Workshop 5	M 19 3 h	Trial and evaluate	DL IT SC2	Video (9.5 h) Photo Documents Field-note
Workshop 6	M 21 3 h	Trial and evaluate	DL IT SC2	Video (9.5 h) Photo Documents Field-note
Analysis M19–24				

In both parts of the intervention proto-typing sessions and participant workshops were documented by three InTouch researchers. Roaming video cameras were used to enable the researchers to move with and occasionally ‘feel’ with the participants, to gain insights into their experiences and probed them to articulate their design process. Whole group discussions were video-recorded. A total of 14 h of video data were collected.

The video data was supplemented by researcher observations in the form of written notes and photographs taken during the workshops and post-workshop field-notes. This included photographs of student prototypes and discussion notes. Cohort 1 participants created digital coursework at the end of the module and these were collected, this included project concept boards (64 PDFs) and group concept videos (12).

The design workshop discussions and activities were documented using post-it notes, flip charts, field-notes and photographs. Finally, the design lecturers’ insights from the workshops and tutorials were documented through the workshop discussion notes and emails, and provided an additional layer of reflective data.

### Analytical framework

**Fig. 2:**
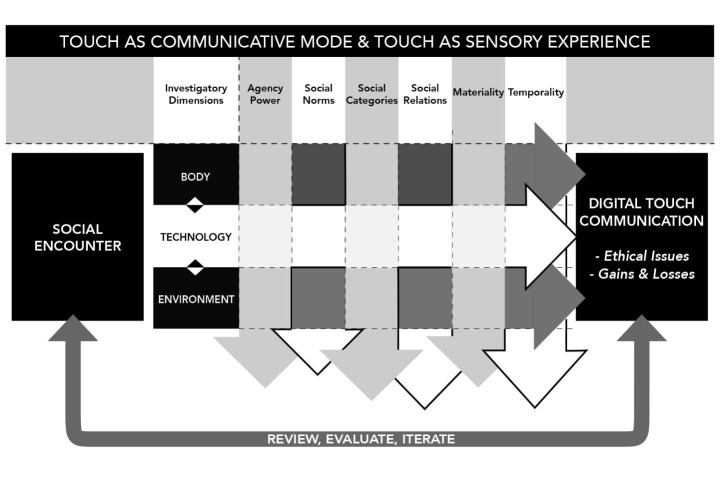
The social, multimodal and sensorial foundation that underpins the analytical framework

The over-arching ‘frame of attention’ that structured our analytical process articulates a simultaneous concern with touch as a communicative mode, and a sensorial experience (Fig. 2). This draws on two inductive data-driven approaches, multimodality (Kress, 2010; Jewitt, Bezemer and O’Halloran, 2016), and sensory studies (Pink, 2015). Although these approaches differ in their emphasis on the semiotic and experiential, and entail different researcher dispositions (e.g., researcher as observer vs. co-participant), they share a qualitative attention to social dimensions of meaning beyond language, place interaction at their centre, and account for that which is shown or felt (Jewitt, and Leder Mackley, 2019).

The framework provides a set of socially oriented investigatory dimensions for the analysis of the participants’ design processes (a social encounter) including agency and power—who or what touches; the social norms of touch; social categories related to touch (e.g., gender); social relations realised through touch; the materiality of touch; and tactile temporalities. These investigatory dimensions are understood as produced through situated social encounters in which the materiality and sociality of the body, the environment and technologies are key interconnecting concepts, providing a second analytical path through the data. The above framework provided multimodal and multisensorial entry points to analyse participants’ design prototypes (i.e., their concepts boards and videos) from part one of the intervention and their interaction with the toolkit in part two.

How this framework was operationalised in part one and two of the intervention is outlined in the relevant sections (see 3. 1.4 and 4.2.3).

## Part one: developing a UXD module on digital touch

### Approach and implementation

Part one of the intervention re-orientated the UX Design module to engage with touch.

Participants were recruited for the study (see 2.3) from the User-Experience Design (UXD) module of the BA Industrial Design and Technology, all 64 students registered on year-two (18 female, 46 male) volunteered to participate in the study. This year-group was selected to ensure a shared understanding and competency in UX design.

A design-brief was co-developed to stimulate innovation on digital touch and explore novice designers’ notions and imagined futures of digitally mediated touch. It instructed participants to “develop an innovative, future-facing digital product or service that enhances communication through touch in one of three sectors: personal relationships, leisure, or health and wellbeing. To first research a specific communication context that would benefit from the introduction of touch technology, for face-to-face or remote interaction; to identify specific user needs and, in collaboration with target users, develop and refine a product or service that will respond to those needs that includes an element of digital touch.” The brief included the following constraints: the design must address a real-world problem, define a target user group, incorporate touch into the design experience that goes beyond touch screens, and reflect on ethical considerations (e.g., safety, wellbeing) and appropriate contexts and boundaries for touch.

Participants attended an introductory lecture (45 min) which situated the brief within existing or emerging technologies, trends and ‘weak signals’ for digital touch, and encouraged them to consider the sociality and sensoriality of touch and the potentials of digital mediation to change touch practices and experiences.

**Fig. 3:**
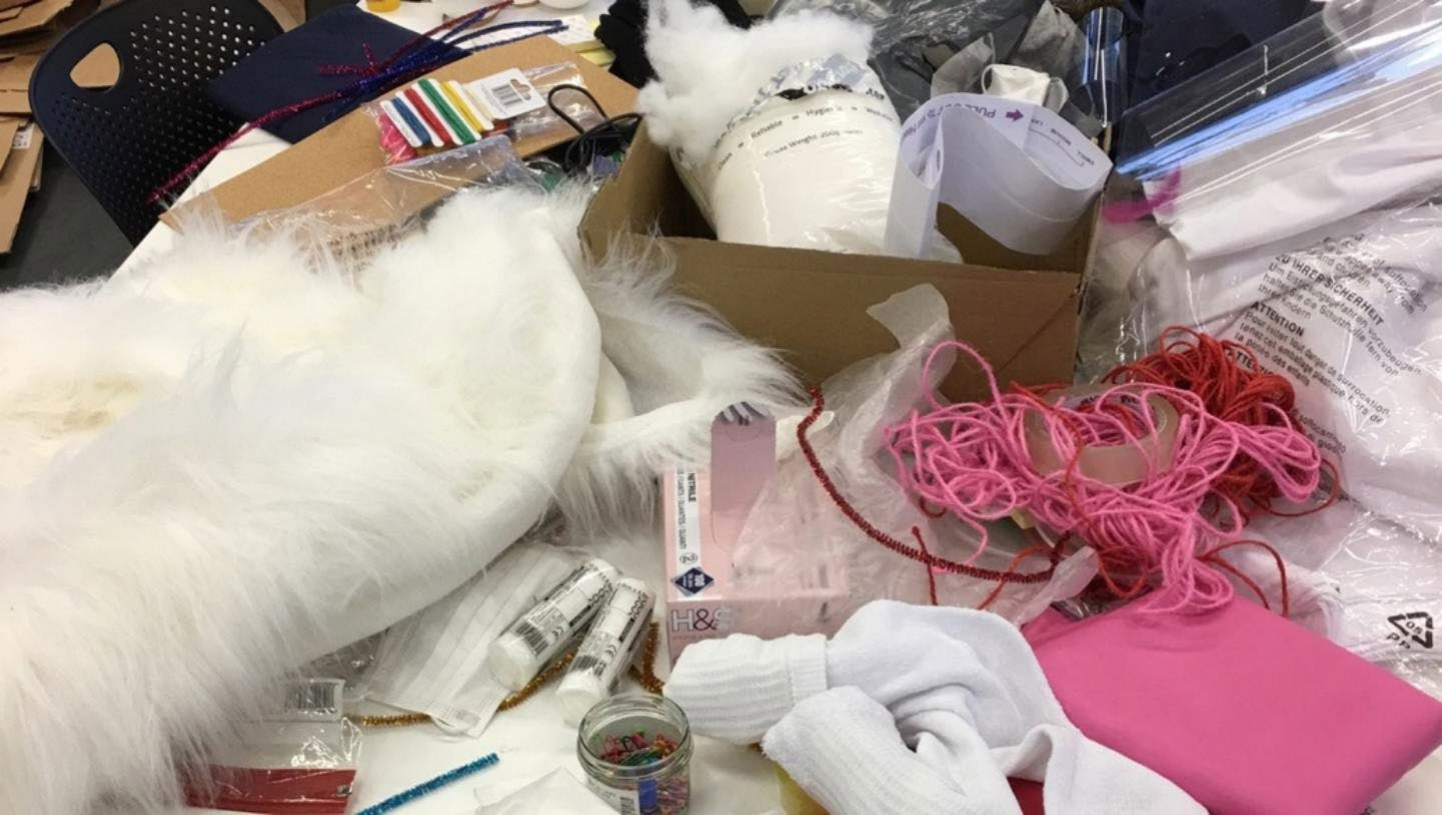
Sensory materials used within the workshop

Two half-day (3.5 h) low-fidelity experience prototyping workshops were facilitated to explore participants’ understanding and conceptualisation of the capabilities of touch in the context of experience design, and the challenges they experienced working with touch in the design process. Workshop one supported participants to generate and refine concept ideas, while workshop two focused on their development of experience prototypes. The participants worked in 10 groups (of 5–7). The first part of the workshop (75 min) included a short introduction; two quick-fire brainstorming activities to sensitize participants to touch as part of everyday experiences, how it might be enhanced; a sensory tour. The tour pointed to the significance of materials as a creative starting point for prototyping, and that design education could usefully “transition from a culture of ‘imparting knowledge about materials’ to a culture of ‘generating experience with materials’” (Pedgley, Rognoli and Karana, 2016). It provided a range of materials, ‘*Body scaffolding’* materials (e.g., white socks, catering hats) (Fig. 3) and a wall display of A–Z touch words.

**Fig. 4:**
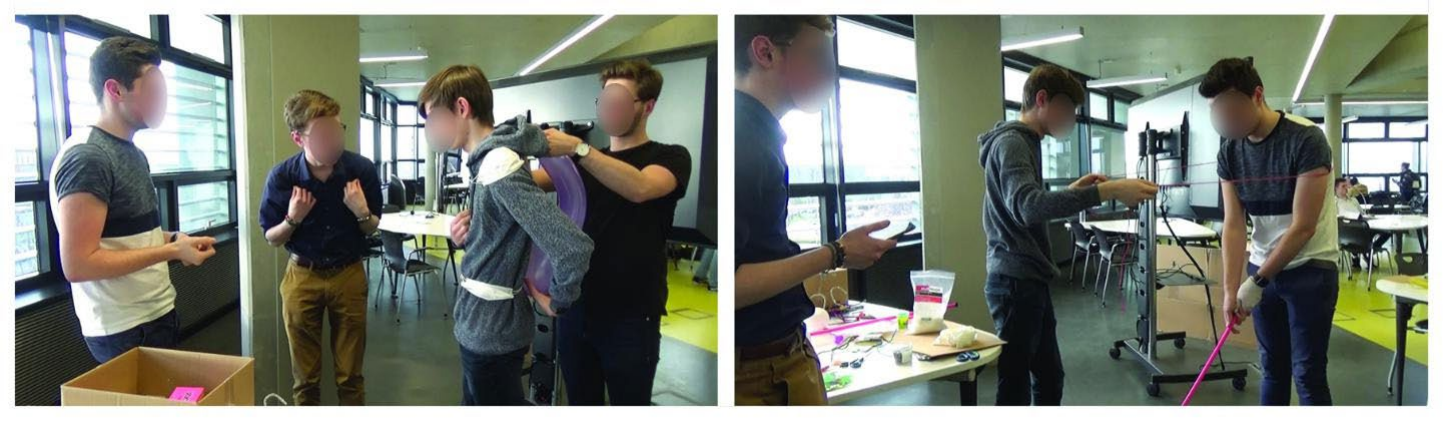
Cohort 1 student group work in the prototyping workshop

The second part of the workshop consisted of low-fidelity experience prototyping—a core pedagogic method used to scaffold ‘*learning by doing*’ and support cycles of experimentation and reflection (Nilson & Dewey, 2006). The prototyping session (90 min) guided participants towards the construction of meaningful narratives to convey emerging touch experiences and empathy with user experiences (Fig. 4). The workshop closed with each group presenting their prototype, a Q& A session, and discussion (30 min).

The researchers observed and video recorded the participants interaction in the workshops and collected their concept boards and videos (see methods 2.4).

The analytical framework (see 2.5) was used to collectively analyse the participants’ concept boards (64) and videos (12) and generated a set of first-order codes. These included the social roles ascribed to touch, the social norms of touch realised, the extent to which the design supplemented, amplified, extended or reconfigured existing touch practices, the types of materials and technologies (e.g. mobile phones, fit-bits) that shaped participants’ prototype designs, thematic areas of interest (e.g. issues of activation and feedback), the settings and relationships that participants associated with touch, the features, affordances and sensorial qualities of touch included, how touch was related to the other senses, the bodily location of touch, and the ethical issues raised. These codes were further explored, clustered and refined through a review of the video and supplementary data to identify key interactional moments. Through this process of working across the data set six higher-order challenges (themes) were developed on how participants took up, and negotiated the design challenges and possibilities of digital touch design, each is outlined below.

### Findings and discussion

The findings from part one of the intervention are organised around six challenges presented below.

#### Identifying a ‘problem’ space for touch in the design process

Touch came in late and was often used to solve an existing problem, raising questions of where and when touch could usefully be a part of the solution space, as a consequence touch was often an awkward add-on. Overall, the participants’ design of digital touch served to mimic, supplement or amplify existing touch experiences, rather than to extend or reconfigure these. The design process often got ‘stuck’ on touch as vibration, and notions of efficiency, convenience, control or feedback. When a solution space was found for touch, it tended to be highly functional, often related to reducing risk (e.g., in relation to the elderly ‘falling’, cycling, accidents and injury) or the maintenance of established relationships in domestic (e.g., home) and public venues (e.g., restaurant).

#### Locating touch on the body

The vulnerability and socially regulation of the body in relation to touch resonated across the participants’ interactions through the design process and outputs. Over a half of participants located touch on the arm, hand or finger; although some extended touch to socially ‘low risk’ ‘accessible’ body touch zones (e.g., shoulder) or the face. When the whole-body was brought into the design process it relied on vibration as a tactile corrective or promoter of kinaesthetic awareness, that is, digital touch was designed to discipline bodies into specific positions or movements. Participants situated touch through the design process in relation to idealised (fit, healthy, able bodied) normative bodies. Wearables dominated participants’ design concepts indicating the conceptualisation of the body as a future touch interface.

#### Moving beyond touch stereotypes

It was a challenge to move beyond often clichéd and stereotypical utopic and dystopic visions of digital touch. The participants’ design processes highlighted their difficulty in engaging with the sociality of digital touch or moving beyond the constraints of dominant contemporary digital forms. The technologies and digital features of participants’ concept designs were bounded by existing technologies and materials (e.g., mobile phones), often limiting digital touch to forms of vibration or functional aspects of activation, feedback, and sensing.

#### Engaging beyond the touch screen

Engagement with the broad character of digital touch was limited, often tied to the ‘screen’ as reimagined on the body, with buttons and alerts a constant feature. Participants rarely moved beyond standard digital touch forms—swiping, tapping, vibration or the use of touch as activating a feature—touch as alert, feedback, connection. Throughout the design process participants did not engage with the sociality of touch—touch etiquettes or social norms, nor the politics or ethics of touch.

#### Engaging with touch communication

Participants rarely explored or engaged with touch as communication in the design process, where communication was brought into focus this related to technological considerations. While prototyping offered participants opportunities to explore the practicalities of receiving and responding to digital touch, there was limited reflection on the communicational potentials digital touch affords (e.g., its temporality, spatiality, and share-ability) and participants drew directly on their experiences of mobile communication (e.g. managing response times). Participants found it difficult to communicate about touch, their descriptive language to narrate touch experiences was limited, and metaphors and analogies often failed -a point made by social science, computer science, and engineering researchers (Obrist, Seah, Subramanian 2013).

#### Touch in the realm of the senses

How to work with touch in relation to the other senses was another design process challenge. Participants primarily engaged with touch as input or output, and relied on the visual, and aural senses, with touch or ‘near touch’ (e.g., a device to alert a cyclist to the proximity of a car) being ‘translated’ into a colour or sound.

The above themes highlight the significant challenges that novice designers experienced in the process of designing digital touch.

## Part two: the designing digital touch toolkit

Part two of the intervention focused on the iterative development and design of a card-based design resource—the Designing Digital Touch (DDT) toolkit—over a year.

### The designing digital touch toolkit

**Fig. 5:**
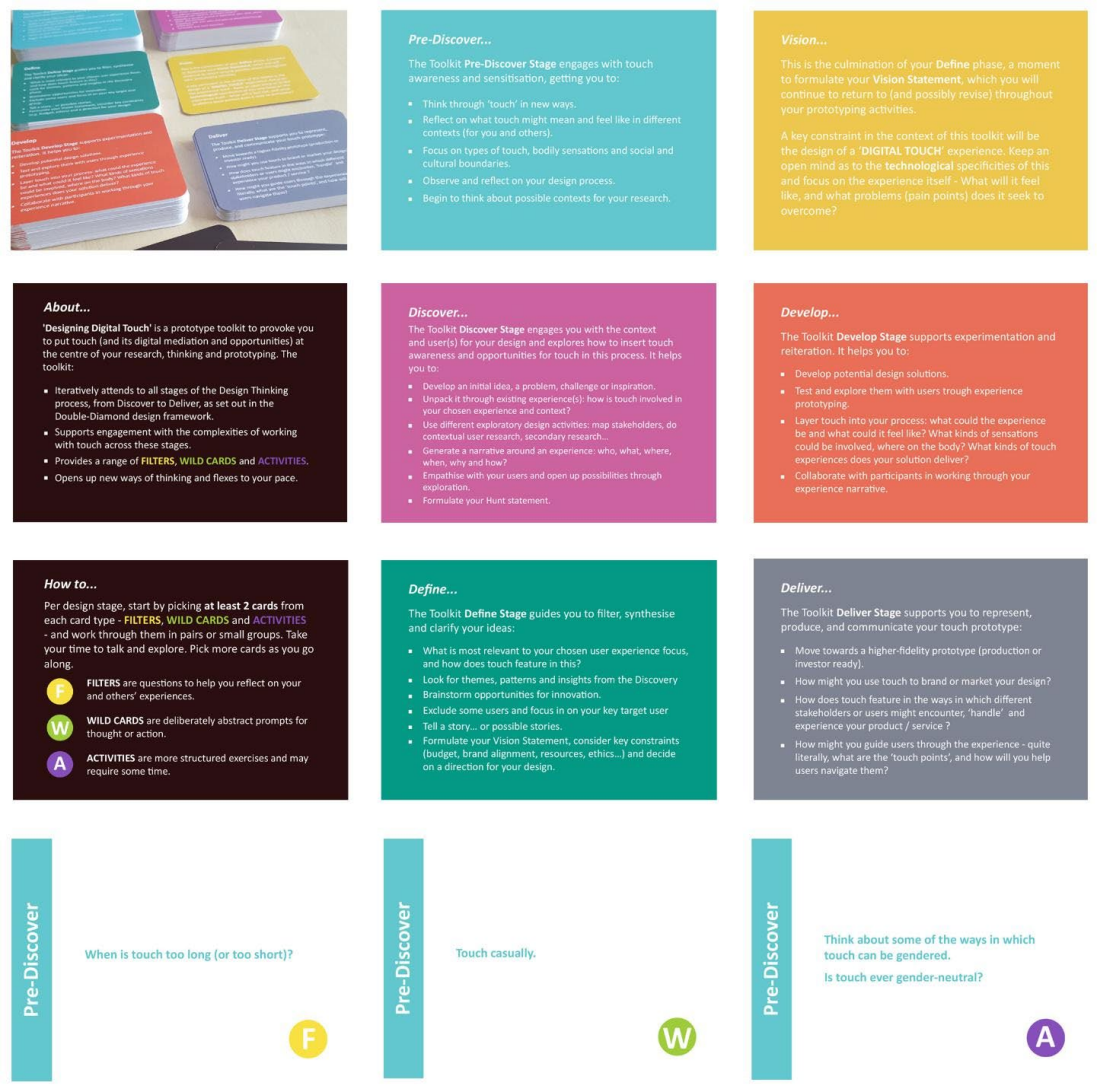
Images of the range of DDT Toolkit cards

The final Designing Digital Touch toolkit consists of 190 cards—9 introductory cards (to explain its purpose, structure and use) and 181 action cards. It is structured by six design stages, which draw on the Double Diamond Design Model (discover, define, vison, develop and deliver), and extend the model through the addition of a pre-discover stage. Three categories of cards (indicated by their initial)—Filters (F), Wild cards (W) and Activities (A), are designed to generate ideas and experiences, probe memories, and provoke imaginations relevant to touch and the design process (Fig. 5). The toolkit cards are designed using Adobe Illustrator and printed using matt colour and one-side lamination (to reduce chipping), on 350 gsm white card, card size 109 mm x 78 mm, and the set is stored in a box.

The toolkit was developed through four design-based workshops, involving cycles of implementation, analysis and exploration, design, evaluation and reflection.

#### Discover

The first workshop worked with the challenges identified in part one to explore ways to support and scaffold novice designers to engage more fully with digitally mediated touch. We returned to the analysis of participant design concepts and prototyping, and selected (based on range) a collection of design scenarios to explore in depth, we reviewed and discussed the participants design process and re-enacted the scenarios in order to identify their sticking points and unpack the challenges of incorporating digital touch, notably its social and sensorial possibilities, into the design process.

#### Define

The second workshop clarified and defined the ‘touch-pinch-points’ identified via the analysis of the challenges and observations of the novice designers’ efforts to incorporate digital touch into their design process. We conducted detailed walk-throughs of participant design concepts and prototypes to refine interventions to support divergent thinking on the design process for touch and we experimented with formats, themes and content for the toolkit cards. This informed the creation of an initial series of prototype resources ‘cards’ (post-it notes) categorized in relation to the stages of the Double Diamond model (Discover, Define, Design, Deliver) and led to the form of the toolkit. The prototype cards were annotated and commented on in relation to direction, structure, tone and content.

#### Develop

**Fig. 6:**
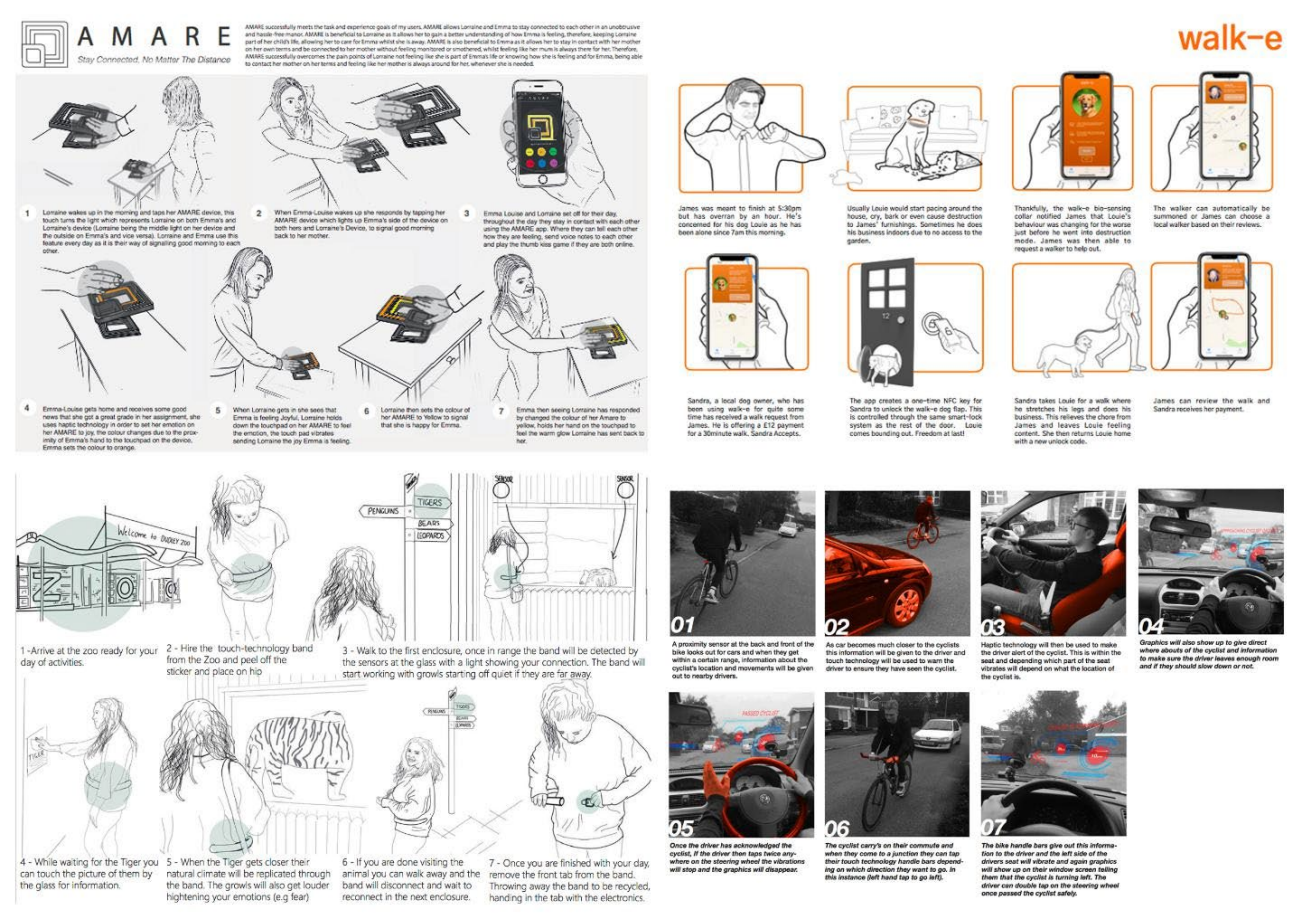
Examples of student concept boards used for the walk-throughs

The third workshop engaged in the iterative development of the structure and content of the prototype-toolkit. We selected a sample of participant design projects and role-played their use by a ‘novice designer’. Design projects were sampled to reflect a range of types of users, use scenarios, touch and technologies: emotional connection (e.g., parent and child); sports coaching (e.g., posture-correcting wearables); physio intervention; safety (e.g., when cycling, skiing, including AR); haptic feedback to enhance Zoo visits; and connect and care for pets, (e.g., bio-sensing collar) (Fig. 6).

We immersed ourselves in the novice designer experience of designing touch through systematic walk-throughs, using the ‘stop-the-action’ technique (Price, Matthews and Wrigley, 2018) to ask questions and experiment with prototype cards and activities. We identified potential gaps or opportunities for touch, critiquing the design process in relation to touch, and interrogating terminology. For example:

[Field-note excerpt—Zoo visit design concept]: We brainstormed what a visitor might want from an encounter with a lion. ‘*A thrill, a rollercoaster experience, fear, excitement?*’ We explored how this might feel and map across the body—‘*heart pounding, stuck fixed to the ground*’. We re-enacted and repeatedly rehearsed this bodily experience, calling out questions to dig into and layer the experience. Once the experience was fully-sketched, we explored how it might be achieved through digital touch technologies. There was a tension between mimicking a physical touch sensation and innovating.

This iterative process enabled us to test, refine and expand the prototype cards and ground them in the work of supporting novice designers.

#### Deliver

The fourth workshop explored how novices might use the prototype toolkit drawing inspiration from other card-based resources and games. We used the toolkit to interrogate an existing digital touch device—the Hey-bracelet. We experimented with the *organisation of the cards*, including a single stack, four stacks separated by design stage, three stacks according to the type of card (F, WC, A); *card selection*, at random or selected; *timed turn taking*, fast, slow or free-time; *interactive mode*, discussion vs. re-enactment or role-play; and *context of card use*, discussion, role-play, design narratives, critical ‘stop the clock’ sessions.

We concluded that keeping the use protocol of the prototype toolkit open and flexible appeared to enhance the users’ ability to respond to the context and specific challenges that arose.

#### Findings and design learnings from the workshops

We identified four main areas of touch that required further fostering and expansion to support the design process of novice designers:

- Conceptualisations of touch as a social, contextual, sensory and physical experience
- Re-imaginations and engagement with the possibilities of digital touch experiences
- Awareness and sensitivity to touch and tactile acuity
- Critical self-reflection and engagement with touch

A list of sub-themes and key areas for a touch design resource was iteratively generated through the workshops. Including the roles of touch, touch qualities, contexts of touch, touch norms, touch temporality, notions of connection, sensing, and the place of touch in emotion, memories and so on. These underpinned the development of the card contents. Through this process three distinct types of resource-cards were developed to scaffold student design processes on touch: Filters (F)—questions to help participants reflect on their own and others experiences; Wild cards (W)—deliberately abstract prompts to open up design thinking; and Activities (A)—structured individual or group exercises. For example, Wild card—Shake someone’s hand—what do you notice? Filter card—What touches are private? What touches are public? Activity card—act out the details of your oldest touch memory?

The need to attune and sensitize novice designers to the physical, sensory and social character and possibilities of touch led to the addition of a *Pre-Discover* phase to the Double Diamond design model.

Cards were developed with attention to fostering self-reflection, imaginative and critical engagement with the tactility of users and use scenarios, and to expand the physical to encompass the sensory and social character and possibilities of touch. Cards to bring the whole body and environment into the design space of touch and to frame touch within a broader multimodal-sensory experience were developed. These aimed to move designs beyond ‘touch equals hand’, fragmenting the body, to place the body in context by exploring the social and sensory environments of touch (e.g., swimming), and body stereotypes (e.g., Filter card—How might touch take account of different bodies?).

The decision to maximize the space for novice designers to consider user ‘experience’ prior to attempting (technological) solutions, resulted in bringing the ‘digital’ into focus later in the design process. Cards were developed to push imaginations beyond touch vibration, functional alert, feedback to a fuller range of touch and to move beyond mimicking or replicating physical touch sensation to consider possibilities for digital touch experiences. Alongside this, cards were developed with a future facing narrative for touch to challenge a technological reliance on existing technologies, and extend the notion of ‘touch-interface’ beyond the mobile screen. For example, Wild card—‘What would digital touch be in a world without phones and touch screens?’, Filter card—‘Could it work without an app? and Activity card—‘find and feel four new sensory experience for your design context’.

Following workshop 4, time guides were added to activity cards, possibilities for active exploration and critique were expanded, content gaps identified and filled. The cards were then collaboratively edited to ensure coherence, check design stage categorisation, and remove repeat or overly ambiguous cards, whilst balancing not being overly instructive. Following a final edit of content wording for sense, the toolkit was printed.

### Toolkit piloting and evaluation

#### Approach and implementation

The Designing Digital Touch (DDT) toolkit was piloted with a second cohort of participants to evaluate and inform its development. Twenty-one full-time MA User Experience Design (MAUXD) students (14 female, 7 male) were recruited via an introductory lecture (as in part one), an introduction to the toolkit, and invitation to two (optional) half-day (3.5 h) experience prototyping workshops. Participants were self-selecting volunteers.

The workshops provided participants with an opportunity to use the toolkit to explore touch in the context of their individual major design projects (rather than in relation to the design brief developed in intervention one). In the first workshop, working in small (4–5) self-selected groups participants outlined their design project direction to each other and then used the toolkit Pre-Discover and Discover cards to explore it in depth in relation to touch. In the second workshop attention was on the Develop, Define and Deliver stages of the design process. Participants first identified and mapped the ‘touch points’ in their design project and created a touch-storyboard, which they discussed in their small groups. The groups were then paired, each chose one design narrative to explore, and took turns to re-enact it using the toolkit cards to ‘stop-the-action’ to collectively interrogate touch. Cards were picked at random—participants found this “more challenging” (the cards they passed on or found confusing were noted). Minimal intervention and probing from facilitators ensured the Toolkit was ‘doing the work’. Both workshops closed with a discussion of the participants’ experiences and critique of using the cards. As described in the methods Sect. (2.4) the researchers observed and video recorded the participants interaction in the workshops.

The framework (see 2.5) was used to analyse the video data of the workshops. This involved viewing the video recordings (as a team and individually), to identify and transcribe (speech and actions) significant moments related to touch and digital touch that unfolded between participants, or between them and materials/ prototypes. Alongside analytical engagement with the fieldnotes and other supplementary materials, this process generated a collection of initial codes. These included touch memories, metaphors, everyday touch narratives and experiences, touch and emotion, touch in relationships (including power), touch norms (e.g., gender) and practices, categorisations of touch, touch design-inspirations (e.g., animals), touch challenges and taboos, the functions and roles attributed to touch, the materiality of touch, valued or problematic touch, and moments of touch awareness/reflection. We also noted how touch featured in the participants interaction, and how this was negotiated between the participants as a resource for exploring touch. These codes were refined and clustered to develop six higher-level themes that encapsulate the participants’ engagement with the design challenges and possibilities of digital touch design (outlined and illustrated below).

#### Findings and discussion

The findings are organised in relation to six key themes.

##### Sensitising participants to touch and digital touch design

The toolkit provided a route into an expanded view of touch and fostered consideration of its sociality including touch norms/etiquette, metaphors, memories, feelings of connection and non-human touch. The cards legitimated and facilitated exploration of a wide range of touch topics including processes of learning touch social rules; personal touch narratives; changes in the social acceptability of touch; hygiene and disease; and sensitive conversations across cultural and personal experiences (e.g., touch preferences). For example, the Filter card, ‘What kinds of touch are private, what kind of touch are public?’, prompted participants to explore where they locate the boundary between these, and differing socio-cultural norms involved.

[G2 participants]

P1: Hugs is private

P2: PDA [Public Displays of Affection], kissing in public

P3: You don’t kiss in public it’s part of [Asian] culture

P1: If it’s a small peck that’s ok?

P1: Putting make-up on the tube…I would feel uncomfortable…it’s so dirty

P2: But it’s okay in a car, we feel that they are private but everyone can see in!

P1: In the UK, I have seen it on the train…

P2: If I tripped on the stairs and someone touched me to steady me, I’d say ‘thank you’, but if someone just reached out to touch me…[recoils] but it’s the same touch’

P1: Curly hair! Touching hair! Strangers don’t even ask they just touch it, it’s very uncomfortable, that’s unwelcome touch!

P3: Its cultural, in Thailand to touch someone you don’t know is very impolite

P1: Touching women’s pregnant bellies, children and dogs!

This card prompted discussion of the importance of context for touch, the rehearsing of ways of asking consent to touch which participants (and the lecturers) considered valuable to foster an understanding of the potentials of touch for experience design.

##### Generating categories of touch

The toolkit encouraged exploration of touch categories and provided a springboard for participants to generate their own categories and continuums, and in the process to find ways to name touch:

P1: [Reads aloud the Pre-Discover Filter card] ‘Who touches as part of their work?’… Masseuse! [hand-motion of giving a massage]

S2: Everyone! Butcher [‘chopping’ hand gesture]

S3: Your right everyone touches!

S4: Cook, Doctor, Nurse…

S1: Maybe we can divide it? People who touch people, and people who touch objects. If you work on a computer, you are touching the keyboard. Maybe touching specifically, like an expert?

S2: You mean the touch related thing has to be expert?

S3: It seems like you have to express something with touch? Like doing [mimics throwing a pot on wheel], and hand-made, making things [small repeated crossing gesture with fingers]

S1: Knitting?

S3: [Nods in agreement]

S1: An expert touch

S4: Teacher, a human touch

S1: Can teachers touch students?!

S2, S3, S4: Laugh loudly for a minute

S3: At least kindergarten teachers should touch students

This group generated four touch categories on a continuum, every-day to expert, artistic to technical which contrasted “*artistic and expressive*” touch with “*more technical like an engineer* “, human to non-human touch, and “*natural touch—gardener, explorers*” which they contrasted with ‘*man-made*’ touch. Other participants generated continuums of touch including welcome-unwelcome, appropriate-inappropriate, childhood-adult touch: fostering engagement with the politics and power of touch.

##### Prompting critical thinking and engagement with complexity of digital touch

The toolkit brought the complexity of touch to the fore through a focus on social and experiential aspects of touch and factors that impact touch (e.g., gender, culture). For example, in workshop 2, during a role-play of a concept concerning ‘Car-VR’- an immersive experience to support the first-time car buyer prior to visiting a car showroom the participants were discussing touch feedback on the feel of the car:

S10: [Reads aloud the Develop Filter card] ‘Is your touch gender-neutral?

S11: Yes?

S12: [Laughs] Do you want to expand on that?

S13: I’d say so—it’s a CAR, so?

S10: Um, aren’t cars, they feel the same to …

S10: I think it would be gender neutral simply because the experience is to reflect as accurately as possible the feel of what the car is like, to inform the buyer

S12: But, do women prioritise different materials or different parts of the car?

S11: What parts they want to touch do you mean?

S12: Yeah, what they want to feel from a car, what kind of emotive language would they be using, what they would want to feel comfortable in it?

S11: The danger is that we do not want to create different experiences for different people if it doesn’t accurately affect their buying experience

S12: I think the idea is that in the buying experience they might be looking for something else?

S13: In CAR-VR would the seats and the materials change? Or is it just the driving simulation?

S12: Yes, if you were having one with a leather finish you could feel that… Maybe if it was a more sporty-car the seats would feel more like bucket seats?

S10: Based on your experience, how much do people care about touch when they are buying a car?

S13: Four out of five car buyers that I spoke to went to car dealers so they could see and feel the car

S12: Touch definitely gives a sense of connection that you can’t get from the internet, because you can’t touch or feel or get a sense of the car space, when you are buying a car, it’s not just about the facts it’s about feeling and touch is a big part of that feeling

S13: You would wear VR-Gloves to feel what kind of material the car is made from?

S10: There would be a very neutral physical steering wheel, pedals and, and the gloves would give the sense of touching materials over that?

The toolkit fostered and supported many such incidences of critical thinking on touch.

##### Designing multisensory and new touch experiences

The toolkit facilitates exploration of touch as a single sense and within a multisensory pallet. For example, the Discover Wild card, ‘Heighten one of the senses, what happens?’ or the Activity card ‘Ask your user to take you on a sensory tour and make explicit how their environment feels, tastes, sounds, and looks’. Participants reflected on how the cards re-orientated their design process to engage with the multisensory character of user experiences. For example, working with the card, ‘How do you touch to evoke emotion?’, they discussed the intersection of touch and gaze, “*A warm touch but blank gaze would be odd and unnatural*”. The cards also *“triggered different ideas*” of the sensory. For instance, the Wild card ‘Touch like an animal’, inspired one group (G5) to consider how they might digitally augment touch:

P20: Cats have whiskers for touch, some use body parts that we haven’t got, would I want that, do I want whiskers, do I want a tail?

P18: I’d quite like a tail.

P21: You can express yourself with a tail.

The group reflected on how this opened up design potentials for touch:

“We are gaining empathy…trying to be like an animal took you out of being a human to think how other beings touch”. (G5, P20)

Participants commented the toolkit offered them new touch vocabularies (e.g., non-human touch), and “*some direction’*, and helped to make “*touch more tangible*”.

##### The body, environment and touch

The toolkit cards provided a reflective space for participants to engage with their own experiences of body, environment and touch as pathways toward critical design consideration of the relationships to touch that their design concept offered. For instance, the Develop Wild card ‘Secret touch’ helped participants reflect on touch newly:

[G1, P1 reads the Develop Wild card aloud, ‘Secret touch’, slowly, her design concept—a system for monitoring pregnancy] “*Feeeels like touch is out-side of our b-o-d-y*. [holds, strokes her stomach] *But it’s i-n-s-i-d-e as well. The baby is inside and that is touching, feeeeeling the baby inside and outside touch*”. She creates a design narrative of connection before and after birth, using sensors to provide the user a sense of ‘*touch inside*’ and “*Touching baby through the pram, rocking and vibrating*”.

‘Secret touch’ inspired others to explore the touching environment. For example, a participant (P6, G2) whose project supported moving to a new city considered that “*wind and air feel different, colder or warmer, torrential rain*” and this led her to add a sensory tour to map a new touching environment to access the sensations of relocation. In these ways, the toolkit supported exploration of alternative directions for the design process, and embedded digital touch in potential user experiences.

##### Exploring touch through touch

**Fig. 7:**
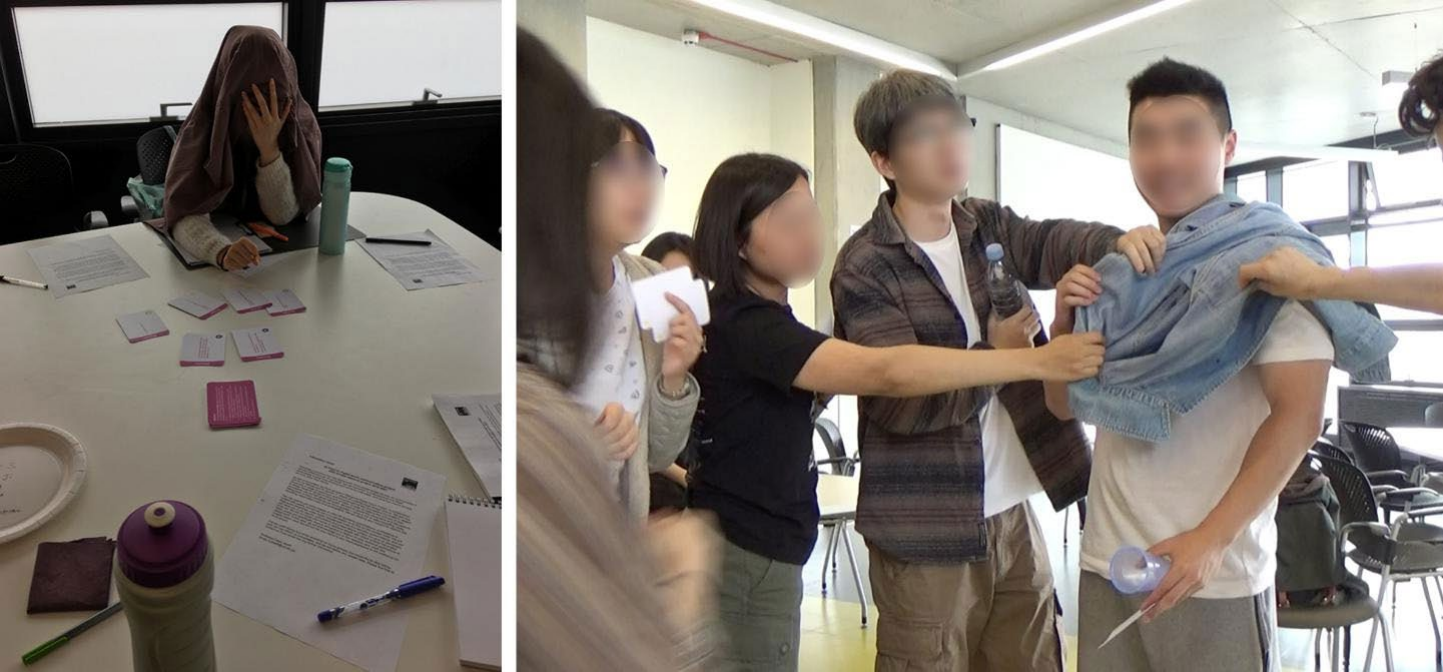
Participants in G3 working with activity cards: (**a**) Discover: ‘Act-out a key moment without sight’ (**b**) Develop: ‘Change its texture’

The toolkit facilitated participants’ interrogation of touch by providing opportunities in the design process “*to flesh out lived experiences*”. Activity cards were key to directing and legitimating participants bringing their bodies and touch into the design process. These cards got participants up and moving, engaged with materials, objects, their self and one-another, and re-enacting touch scenarios. This supported participants to focus, re-imagine and critique the felt potentials of touch for design (Fig. 7). In the context of developing the ‘Memory Jacket’, a wearable design concept (P9, G3), for example, using the cards to ‘stop-the-action’ and interrogate the scenario through touching, prompted new questions related to the recording, storing and playing of tactile memories.

P6: [Reads aloud the card]: ‘Change the texture’.

P9: I think it could not only be the jacket, but also the skin, sensors that feel the skin, that assess the environment, maybe think ‘out of the jacket?’

P8: What’s the texture of the jacket now? [They feel it]

P7: Or is this about the texture of the feeling?

They imagine it sweaty and damp and creating felt experiences through temperature, moisture, the sensation of the heart-rate, and recording touch experiences.

P6: I don’t want to put it on! If the function of the jacket is to give me the feeling, I don’t want to change it, the smell and the feeling of it.

Responses to Activity cards that involved participants touching one another varied: some engaged enthusiastically, others experienced some moments of discomfort met with ‘contactless’ re-enactment, self-touching, comment or quietly passed-over. For example:

(G5) Turned over Wild card ‘Touch a human’. They ignore the card and, without comment, take another. A few minutes later, one participant says “*That’s funny, it says touch a human and not a single one of us has touched a human*”. They laugh loudly. Smiling, a participant gently touches another’s shoulder with one finger, they all do this, and laugh more.

Participants explored and developed ways to manage the ethics of touching raised through this touchy design process including tacitly agreeing to discuss rather than enact touch, only touching friends (pointing to the importance of self-selected groupings), displaying sensitivity to cross gender touching, and routines to request/check consent prior to touching.

The toolkit raised ethical questions for the authors as researchers/designers: ‘*Is it okay to ask them to touch one another?*’. We acknowledged that social and cultural norms of touch are always in play, and that some of the cards did not, could not, and perhaps should not adequately legitimate or support touching between the participants. This was significant, as while the importance of human values and ethics is well established in technology and design education literature, it often fails to translate into practical and tangible tools for design students (Kheirandish et al., 2020). By placing the social and the sensorial at the centre of this toolkit and therefore the design process, the toolkit emphasises and strengthens the ethical responsibility of designers with respect to digital touch, an issue that informed the next iteration of the toolkit.

##### Use of the toolkit

The use of the toolkit was most productive under the following conditions—in small groups (4–5) as this enabled touch to be contextualised within the participants design concepts; where discussion could be complemented with bodily re-enactment; card selection was random; turn-taking was rapid; and when used alongside role-play and ‘stop-the-action’ techniques (Price, Matthews and Wrigley, 2018). These conditions helped to generate spontaneous ideas, avoid ‘over-thinking’, deepen participant engagement with digital touch, encouraged (and de-personalised) critical engagement, and supported divergent responses to cards which built a collective confidence to interpret them.

The workshop participants proposed alternative methods for using the toolkit including the use of the cards to build a collaborative design narrative or scenario; as part of a design interview with users “*to help them think through the touch experiences that they might like to have or in relation to your design*” (P14, G4) or to gain insight on what they see as relevant. Many saw the cards’ potential for individual use, as a resource to support focus, generate ideas, and ‘*spark*’ lateral thinking (some photographed them for later use). Participants commented positively on handling the physical toolkit and imagined possibilities for touch interaction with a digital version, “*you could shake it for a wild card*” and include a ‘*shuffle*’ option, noting its lower-cost and ease of access as advantages.

### Toolkit iteration and refinement

The analysis of the pilot workshops with novice designers informed a further iteration of the toolkit to amplify its strengths and reduce its weaknesses. All cards were reviewed by the team and the following changes made to:

- Better sensitize novice designers to touch
- Foster an openness beyond a functional approach to touch
- Extend touch vocabularies as a springboard for discursive exploration and design
- More clearly situate touch through a focus on context
- Enhance opportunities for reflection and critical thinking
- Promote empathetic repositioning to enable switches in perspective on touch
- Expand possibilities for active exploration through touch in the design process
- Re-calibrate the cards to better balance clarity with ambiguity
- Extend engagement with the ethics of touching
- Clarify time-guides for Activities

Repetitive cards and cards that had not resonated with participants were removed. New cards were added in response to the gaps identified through the workshops (e.g., ‘mouth-feel’). The cards were edited once more for clarity and conciseness.

Finally, although outside of the scope of this paper, the toolkit was digitalised as a free online resource: https://www.in-touch-ucl.design. This overcomes the limitations of access and durability to physical toolkits raised by the participants and other studies (Roy & Warren, 2019) (and concerns regarding hygiene raised by Covid-19). While the toolkit contents remain the same there are two significant differences between the physical and digital version: first, the digital version includes a ‘shuffle’ option (as suggested by the participants); second, users can entirely by-pass the stages of the Double Diamond Model by searching or shuffling ‘all cards’. Further research is planned to explore the take up and use of the digital version of the DDT Toolkit.

## General discussion

The potentials of technologies for the digital remediation of touch and its extension or reconfiguration as a communicative sense are highlighted by the literature, however, this paper has shown that novice designers can be ill-equipped to fully grasp these. While the development of an UX module on digital touch design (i.e., part one of the study intervention) offered a degree of scaffolding to support novice designers’ to engage with digital touch design—framing the design problem, offering information, design strategies and methods to work with touch (Björklund, 2013; Deininger et al., 2017; Osmond, Bull & Tovey, 2009), our analysis shows that novices continued to experience significant challenges in the design of digital touch. The study identified and described six major challenges that the novice designers faced when working with digitally mediated touch. These related to clarifying a design-space for touch, locating touch beyond the hand, moving beyond utopic, dystopic or stereotyped visions of digital touch, a lack of social sensitivity and critical design perspective on touch, difficulties getting beyond the technological considerations, and the challenge of balancing touch in relation to other senses. These findings resonate with the complexity of designing touch, especially given its tacit status in design processes (Schindler, 2015), the difficulty of talking about tactile experiences (Obrist, Seah and Subramanian, 2013), and the ways that technologies can fragment the body (Jewitt, Xambo, and Price, 2017). While the current state-of-the-art in haptic technology showcases the efforts of designers to make creative use of technologies, touch remains a complex space for technology and design to mediate (Jewitt et al., 2020; Parisi, Paterson and Archer, 2017). Part one of the study highlighted the need for additional design resources to foster and support novice designers entering this complex space.

In response the DDT toolkit was developed to make the phenomena of digital touch and its design more tangible (Brandt and Messerter, 2004). The paper mapped the process of its development and evaluation. Findings showed the potential of the DDT toolkit to support novice designers, notably by linking touch-based and bodily-physical activity with divergent design thinking to help scaffold the design process (Hu et al., 2019). We demonstrated the ability of the toolkit to facilitate, externalize and communicate in the design process (Sanders, 2005); to provide information at levels relevant to novice designers (Deininger et al., 2017); to help generate touch vocabulary, metaphors, and typologies to scaffold discussion and overcome the difficulties of talking about touch (Obrist, Seah and Subramanian, 2013); to prompt critical questioning of assumptions and broaden the types of questions asked about touch; to foster reflection and engagement with the sensory and the social aspects of touch; and to facilitate imaginative speculation and creative thinking towards alternative futures—which Gradwell (1999) argues are central to technology education design. In short, we showed how the toolkit can support novices’ systematic engagement with the complexities of an expanded approach to touch and the design of digital touch.

The study methodology has three main limitations. First, the novice designers who participated in the study were university design students. Whilst acknowledging this limitation, the studio as an academic entity accounts for the larger disciplinary community of practice and seeks to provide a bridge “where students can learn the norms, practices, and tools use of the larger professional community of practice” (Brandt et al., 2013:346), and the emergent character and state-of-the-art of digital touch design suggests that while novice designers in other design contexts would face similar challenges. Second, the study is situated within UX Design which is associated with specific histories and practices (Mitchell and Melinkova, 2018). However, the range and flexibility of the toolkit and its extended approach to touch, we argue that it can accommodate differences in experience, giving the toolkit purchase for design more generally. Third, the toolkit’s structure being entangled with the design stages of the Double Diamond Model could be considered a limitation by some. However, the model is infused through a broader iterative human-centred design process, the social and sensorial aspects of design, and the speculative character of digital touch design and, as noted earlier, the online version of the toolkit enables users to entirely by-pass the stages of the Double Diamond Model. The toolkit can, therefore, be used within a variety of design models and theories.

## Conclusions

This paper has presented a qualitative DBR study centred on a two-part educational intervention on the design of digital touch within a UK design school. It sought to narrow the gap between the promise of technologies to remediate touch and the challenges novice designers face when designing digital touch. Part one involved the development and delivery of a UX design module with a focus on digital touch, and investigated how novice designers approached the phenomena through a series of activities. The study findings from part one provided insight on the challenges that novice designers faced when integrating touch into their design process. Grounded in and motivated by these findings, part two of the intervention focused on the iterative development, testing and design of the Designing Digital Touch toolkit, a 190 card-based design resource. The decisions involved in the development of the toolkit were outlined, and the findings from the toolkit piloting and evaluation showed its potential to support novice designers with information, awareness and inspiration via novel routes to engagement with the social, sensorial and the future-facing complexity of digital touch design.
